# Reality Check of Laboratory Service Effectiveness during Pandemic (H1N1) 2009, Victoria, Australia

**DOI:** 10.3201/eid1706.101747

**Published:** 2011-06

**Authors:** Michael Catton, Julian Druce, Georgina Papadakis, Thomas Tran, Christopher Birch

**Affiliations:** Author affiliation: Victorian Infectious Diseases Reference Laboratory, North Melbourne, Victoria, Australia

**Keywords:** pandemic (H1N1) 2009, laboratories, laboratory techniques and procedures, planning techniques, specimen handling, influenza, viruses, Australia, synopsis

## Abstract

In Australia, the outbreak of pandemic (H1N1) 2009 began in Melbourne, Victoria; in the first 17 days, the Victorian Infectious Diseases Reference Laboratory detected 977 cases. Although the laboratory had a pandemic plan in place, a retrospective evaluation found 3 major variations from plan assumptions: 1) higher peak demand not limited by a case definition, 2) prolonged peak demand because containment attempts continued despite widespread influenza, and 3) unexpected influence of negative test results on public health actions. Although implementation of the plan was generally successful, the greatest challenges were limited availability of skilled staff and test reagents. Despite peak demand of 1,401 tests per day, results were provided within the usual 24 hours of specimen receipt; however, turnaround time seemed slower because of slow transport times (>3 days for 45% of specimens). Hence, effective laboratory capability might be enhanced by speeding transport of specimens and improving transmission of clinical data.

## Abstract 2

TOC summary: The greatest challenges were insufficient staff and test reagents.

*No campaign plan survives first contact with the enemy.—*Helmuth Graf von Moltke

The pandemic (H1N1) 2009 outbreak in Australia was detected in Victoria on May 18, 2009, and during the following weeks spread to other states. Pandemic planning guidelines for Australia consist of 4 phases (*1*): delay (identify and test persons who meet a clinical case definition), contain (home quarantine laboratory-confirmed case-patients and give antiviral prophylaxis to their contacts), sustain (restrict laboratory testing to persons with clinically defined cases who are at increased risk for severe outcomes), and protect (identify and manage those at risk for severe illness and those in vulnerable settings such as aged-care facilities). The pandemic plan envisaged all Australian states moving together through the pandemic phases. In practice, however, Victoria implemented the sustain phase, referred to as modified-sustain, sooner than other states.

The first 3 case-patients were siblings who had recently returned from the United States (Figure 1). When the outbreak began, Victorian health authorities implemented the contain phase (*3*), and laboratory confirmation of cases was conducted by the Victorian Infectious Diseases Reference Laboratory (VIDRL). Attempted containment ceased on June 3 when confirmed cases totaled 977, at which time laboratory testing was restricted to that appropriate under a modified-sustain phase. By June 23, when the modified-sustain phase ended, 1,406 cases had been laboratory confirmed and 1 patient had died. Testing efforts subsequently moved to those required under the protect phase. By September 27, a total of 6,895 cases in Victoria had been reported, 24 of them fatal (*3*), although the true number of cases is probably greater.

**Figure 1:**
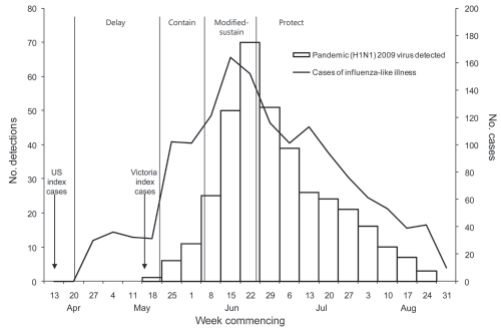
Number of patients with influenza-like illness and numbers of laboratory detections of pandemic (H1N1) 2009 derived from primary care physician influenza surveillance together with the phases of the outbreak in Victoria (VIC). The phases are as follows: delay (conduct active surveillance and border control measures), contain (restrict establishment of the pandemic), modified-sustain (minimize transmission and maintain health services), and protect (focus on those at risk for severe outcomes). Modified from (*1*,*2*),

We describe VIDRL provision of laboratory support for the pandemic (H1N1) 2009 outbreak response in Victoria. We critically appraise the effectiveness of this laboratory’s pandemic planning from 3 perspectives: 1) how the reality of the pandemic matched planning assumptions, 2) how successfully this planning facilitated workflow in practice, and 3) how successfully the laboratory delivered the required testing.

## Pandemic Planning

Our planned algorithm for influenza A virus testing involved extraction of RNA from clinical specimens by using QIAxtractor or BioRobot Universal System extraction robots (each from QIAGEN, Valencia, CA, USA), followed by reverse transcription with random hexamers. cDNA was amplified in parallel assays by using an Applied Biosystems 7500 Fast Real-Time PCR System (Foster City, CA, USA) and incorporating primers and probes selective for the matrix gene of influenza A viruses, including that of the pandemic (H1N1) 2009 virus, and for the hemagglutinin (HA) gene of that virus. (Sequences of all primers and probes used in these assays are available upon request to M.C.).

Our model of anticipated pandemic influenza testing comprised 2 phases. First, an initial peak of intense testing needed to identify early cases would result in >500 additional PCRs being conducted each day for 2 weeks. Second, a step-down in demand with a focus on severe or atypical cases that needed testing for clinical management would result in ≈200 tests being conducted each day for several months. Implicit in the latter phase was that a clinical case definition would suffice for most uncomplicated influenza cases and that dominant circulation of the pandemic strain would enable a test result of “influenza A detected” from many laboratories to be a de facto diagnosis of pandemic (H1N1) 2009 infection. Some laboratory capacity would be reserved for outbreak monitoring by sentinel surveillance and detailed strain characterization. All routine diagnostic laboratory activity (≈1,000 tests/day) for diseases other than influenza would proceed routinely, but elective activities such as research would be delayed as needed.

To realize this pandemic plan, certain measures were undertaken at VIDRL. They were 1) assembly of enough nucleic acid extraction robotics and real-time PCR analyzers for >500 daily PCRs, 2) recruitment and training of 2 additional scientists who could work in the testing laboratory during a major outbreak, 3) planning for the temporary reassignment of scientific staff with appropriate skills from other laboratory areas during an outbreak, 4) cross-training of secretarial and clerical staff to enter patient and specimen data into the laboratory information system, 5) manning of the laboratory telephone switchboard by clerical staff, and 6) creation of a small stockpile of essential laboratory reagents.

## Effectiveness of Testing

**Figure 2:**
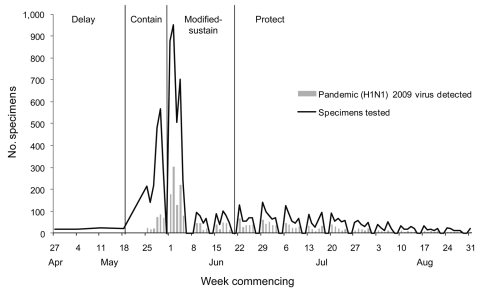
Number of diagnostic specimens received at the Victorian Infectious Diseases Reference Laboratory and laboratory detections of pandemic (H1N1) 2009 virus, Victoria, Australia, 2009.

During the initial contain phase, the number of tests run was high. On June 1, the day of peak testing, 1,401 PCRs for influenza were performed, this being the sum of the matrix gene PCRs performed on each referred specimen and HA gene PCRs performed on matrix gene PCR-positive samples (Figure 2). In contrast, a typical daily peak number in winter would be ≈100. However, the laboratory was able to sustain peak levels of influenza testing and provision of results within typical turnaround times. The times from specimen data entry into the laboratory information system to result reporting were calculated by extracting data from the Laboratory Information System (Medipath, LRS Health; Melbourne, Victoria, Australia) with an integral analytic software module. Because the actual time of specimen arrival is not searchable on our system, the representativeness of this electronic data as a proxy for total test turnaround time was verified by a manual audit of 200 Medipath files. This procedure compared the manually stamped arrival time and date on scanned digital images of specimen request forms received on June 1, the busiest day of the outbreak, with the corresponding time and date recorded electronically for result reporting. This manual audit gave a faster estimate for turnaround time than the electronic search, probably because the latter includes data from weekends (data not shown).

**Figure 3:**
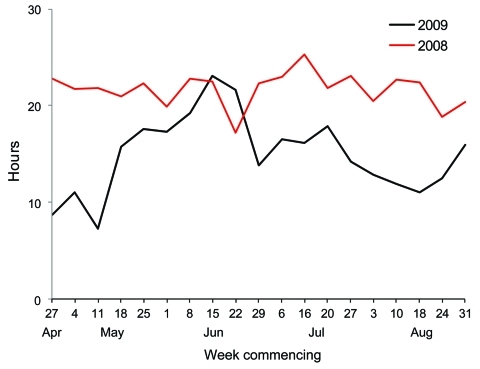
Mean turnaround times for Victorian Infectious Diseases Reference Laboratory detection of influenza, Victoria, Australia, 2008 and 2009.

The mean turnaround time from specimen data acquisition to result reporting for the 4 peak months of the 2009 outbreak was <24 hours (Figure 3). For all except a 2-week period in June, this turnaround time was faster than the equivalent turnaround time for the winter of 2008. The main contributors to this outcome were longer than usual working hours for scientific and support staff, coupled with high levels of automation.

**Figure 4:**
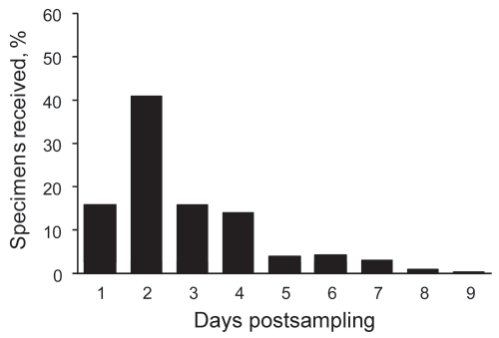
Timing of receipt of pandemic (H1N1) 2009 virus–positive specimens by the Victorian Infectious Diseases Reference Laboratory, Victoria, Australia, 2009.

Specimens were transported by courier to VIDRL from Melbourne hospitals, other laboratories, and general practitioners on behalf of Victorian health authorities. The duration of time from specimen collection to arrival at VIDRL varied. Transport times for all pandemic (H1N1) 2009–positive samples were calculated by comparing the interval between the laboratory receipt time and date stamp and the recorded collection time and date on digital images of specimen request cards. Positive samples were chosen for analysis because of the relative ease with which this dataset could be collated from the laboratory information system. The positive samples were representative of the total sample group from which they came; ≈15% of positive specimens arrived on the day of collection, 40% arrived the next day, and ≈30% arrived over the next 2 days (Figure 4). Despite maintenance of typical test turnaround times, these transport times contributed to clinicians’ perception of slow turnaround times (*4*), for which VIDRL received numerous complaints. During the pandemic, it was common to receive telephone inquiries for results for specimens that had arrived only hours earlier or had yet to arrive.

Our pandemic planning had focused primarily on resources and processes under our control within the laboratory. However, for optimal functioning of the whole testing cycle, the movement of specimens and accompanying data from patient to testing site and provision of results back to the patients’ caregivers must also be optimal. To do so required a systemwide planning approach that was less than complete at the onset of the pandemic. More planning will be needed for optimal functioning under the pressures imposed by a future large outbreak (Table).

**Table:** Summary of laboratory effectiveness during pandemic (H1N1) 2009, Victoria, Australia, 2009

Challenge	Potential solution
Data management	
Pressure on specimen data entry into laboratory information system	Direct electronic communications of specimen data from referring source to laboratory
Missing telephone, fax, address details on request forms	Direct electronic communication of results from laboratory to referring source
Volume of negative results precluding telephone contact with referring source	Direct electronic communication of results from laboratory to referring source
Specimen transport	
Slow	Multi-institution planning of efficient emergency specimen transport
Poor interfacing with test start times in laboratory	Multi-institution planning of efficient emergency specimen transport
Staff	
Finite laboratory staff resources	Further minimization of manual steps for specimen processing and additional staff cross-training
Telephone inquiries	
Difficulty manning switchboard over extended laboratory hours	Planning for additional agency staff during emergencies
High call volume to laboratory taking scientific staff away from testing	Minimization of inquiries through improved specimen transport and data management
Reagents	
Shortages threatening test capacity	Expansion of reagent stockpile and use of validated test protocols using reduced reagent volumes
Communication	
Misunderstandings regarding scope and objectives of laboratory testing	Strengthened lines of communication between laboratories, clinicians, and health authorities
Pandemic planning	
Lack of flexibility to accommodate verging levels of influenza activity at state jurisdiction level	Adapted pandemic plan

## Effectiveness of Pandemic Planning

During the pandemic, 3 key elements differed substantially from our planning assumptions: 1) we did not predict the expectation that all community respiratory disease would be tested, 2) we did not plan for testing to continue long after widespread community spread of influenza was evident, and 3) we had not considered that negative test results would be so influential to the public health response. This outbreak was the first influenza pandemic during which provision of real-time diagnostic virologic testing on large numbers of specimens had been a practical possibility. This testing capability created high expectations among users of our service. Our pandemic planning had sought to provide a realistic volume of testing capacity for anticipated public health and clinical needs. However, the initial expectation from the community and many clinicians during the contain phases was that all cases of respiratory disease in the community would be tested. This expectation is not unusual in highly publicized infectious disease outbreaks, but because the at-risk population was effectively unlimited in this outbreak, the demand was extreme. Most samples received were from persons who were relatively healthy, as evidenced by telephone conversations between our medical staff and patients, clinical details when provided on request forms, and by the dramatic drop in demand later during the sustain phase when testing was focused on those truly at risk for serious illness (Figure 2).

Our planning model of a 2-week initial surge followed by a step-down to clinically focused testing proved correct. However, the contain phase of high-demand testing continued well beyond the point at which it was first evident that community transmission was widespread. Only 9 of the first 978 case-patients had a history of overseas travel (*3*), and pandemic (H1N1) 2009 began to be detected from our sentinel general practitioner influenza surveillance network within the first week of the outbreak (*3*). Unlimited testing as influenza spread rapidly in the community drove testing demand to extremely high levels. The reasons for continuation of the contain phase are complex but were in part a consequence of the pandemic plan’s treatment of the country as a homogeneous whole, although in reality the Victoria outbreak occurred several weeks sooner than outbreaks in other Australian states (*5*). In contrast to the higher than expected peak, testing levels during the subsequent step-down phase were lower than provided for in our plan (Figure 2). This finding is consistent with the relative clinical mildness of the pandemic (H1N1) 2009 virus strain; in Victoria, only 0.3% of infected patients were hospitalized in the first 10 weeks of the outbreak (*6*).

In past outbreaks, we focused on urgent and accurate communication of positive laboratory results that identified cases, and we communicated negative results en masse by routine systems, including electronic links to major health care institutions. However, during pandemic (H1N1) 2009, major public health actions were triggered by negative results, including cessation of quarantine restrictions and decisions about antiviral prophylaxis. While communication of large numbers of positive results to clinicians and public health authorities challenged resources, urgent and personalized transmission of a much larger number of negative results was not possible. This limitation was further compounded by the frequency with which telephone or fax numbers of primary care physicians were missing on request forms; hence, laboratory reporting depended on postal addresses, which were also frequently incomplete or missing. Spot checks of request forms performed several times during the outbreak found this problem on up to 10% of request forms.

## Implementation of Planning

Many aspects of our laboratory pandemic planning worked well in practice; outbreak testing facilities and equipment platforms provided the required test capacity (as many as 1,400 extra PCRs in 1 day). Employment of additional scientists before the outbreak also provided considerable benefits. In other areas, a great deal of commitment and hard work from staff compensated for planning shortcomings. Notably, preparations for surge capacity in several support areas, including patient data entry and dealing with telephone inquiries, could not match demand and required additional effort to resolve bottlenecks. Because our system of data entry requires specific skills, we could not use temporary agency staff for data entry. In practice, cross-trained secretarial staff and volunteers proved too slow for the demand, and their needs for support impeded the work of skilled staff. Particularly after hours, laboratory test results were often available before complete data entry had been performed, delaying release of hard-copy laboratory reports. A technical solution involving electronic upload of test requests from clinicians seems the best future approach to this problem.

Scientists in our organization who were not involved in influenza testing, envisaged as providing a pool of supplementary staff with PCR or virology skills, were rarely able to perform this function during the outbreak. The capacity of support staff who were performing functions such as specimen reception was almost entirely consumed by the demands of receiving influenza specimens. Staff in other laboratory areas helped absorb demand by taking over these functions for their own specimens but then could not reasonably release scientific staff to supplement influenza testing. As a result, those involved in influenza testing worked long hours, supported by scientists from other laboratory areas who were also working overtime. Although this approach was sustainable for weeks, it could not have continued through the outbreak.

Lastly, the small stockpile of PCR reagents proved insufficient. The high demand for testing during the contain phase required a commensurate amount of reagents. Suppliers in Australia were initially unable to keep up with our rapidly escalated demand. This limitation was successfully managed by using reduced reaction volumes (because of a shortage of random hexamers, the volume of reverse-transcribed cDNA was halved); changing aspects of our testing algorithm (from an initial test algorithm involving influenza A matrix gene PCR primers and H1 HA gene primers run in parallel to an algorithm involving the matrix gene alone with subsequent HA subtyping of positive samples on the same day); and, immediately after introduction of the modified-sustain phase, adhering rigidly to the criteria for test eligibility circulated by health authorities. Adhering to these criteria included storing, but not testing, samples from persons determined to not be at substantial clinical risk. This practice caused unhappiness among some clinical colleagues but preserved sufficient capacity to guarantee testing for patients in clinical need.

## Outbreak Monitoring

As described elsewhere (*2*,*3*), a network of 80 general practitioners in metropolitan Melbourne and rural Victoria conducted influenza surveillance, coordinated by VIDRL, from May through October 2009. Laboratory testing for influenza was conducted for a subset of these cases, and test results were made available online (*7*). This testing activity was maintained during the time of heavy laboratory demand because of the perceived need to collect unbiased data on influenza activity comparable to data collected during the previous 10 years of influenza surveillance.

The number of laboratory-confirmed cases of pandemic influenza (*3*) was heavily influenced by community testing behavior and by guidelines for testing promulgated by health authorities. This influence is shown clearly in the abrupt reductions in testing and detections of influenza in Victoria after June 3, when the pandemic response phase changed from contain to modified-sustain (Figure 2). Hence, the number and timing of laboratory-confirmed cases were unrepresentative of the wider outbreak. In contrast, laboratory-supported influenza surveillance undertaken in parallel with diagnostic testing provided monitoring of the course of the outbreak relatively free of these effects (Figure 1) and, as described elsewhere, enabled direct comparison of the outbreak with >10 years of seasonal influenza (*3*,*7*,*8*).

## Conclusions

Operationally, the pandemic (H1N1) 2009 outbreak tested our laboratory preparedness in ways that no exercise could; yet some of the potential pressures were limited by the relatively low clinical severity of the virus. The numbers, speed, and accuracy of tests conducted, along with real-time tracking of the outbreak through laboratory-supported influenza surveillance, were unimaginable less than a decade ago. Facilities, equipment, and PCR-based testing performed extremely well. Limits to the available pool of skilled staff and the threat of reagent shortages provided challenges where contingency plans had only been partly successful. Staff performed admirably in the face of these challenges, but in the future, more effective solutions will be required. The greatest improvements in overall performance of the laboratory testing cycle will be achieved through increasing the speed of specimen transport and improving transmission of clinical data to and from the laboratory.
